# Dihydropyrimidinones Against Multiresistant Bacteria

**DOI:** 10.3389/fmicb.2022.743213

**Published:** 2022-03-18

**Authors:** Marisa Castro Jara, Allison Carlos Assunção Silva, Marina Ritter, Adriana Fernandes da Silva, Carolina Lambrecht Gonçalves, Pedro Rassier dos Santos, Luciano Sisconetto Borja, Cláudio Martin Pereira de Pereira, Patrícia da Silva Nascente

**Affiliations:** ^1^Postgraduate Program in Biochemistry and Bioprospecting, Federal University of Pelotas, Pelotas, Brazil; ^2^Department of Microbiology and Parasitology, Institute of Biology, Federal University of Pelotas, Pelotas, Brazil; ^3^Lipidomics and Bioorganics Laboratory, Center for Chemical, Pharmaceutical and Food Sciences, Federal University of Pelotas, Pelotas, Brazil; ^4^Cell Culture and Molecular Biology Laboratory, Federal University of Pelotas, Pelotas, Brazil

**Keywords:** antibacterial activity, biginelli compounds, cytotoxicity, DHPM, dihydropyrimidinones, hospital infection

## Abstract

The increase in bacterial resistance to antimicrobials has led to high morbidity and mortality rates, posing a major public health problem, requiring the discovery of novel antimicrobial substances. The biological samples were identified as the Gram-negative bacilli *Acinetobacter baumannii*, *Escherichia coli*, *Enterobacter cloacae*, *Klebsiella pneumoniae*, *Morganella morgannii*, *Pseudomonas aeruginosa* and *Serratia marcescens* and the Gram-positive cocci *Enterococcus faecium*, and *Staphylococcus aureus*, all of them resistant to at least three classes of antimicrobials. The antibacterial activity of the compounds was checked *in vitro* by determining the minimum inhibitory concentration (MIC) and minimum bactericidal concentration (MBC) by the broth microdilution method and plating in brain heart infusion (BHI) agar, respectively. The chemical characterization of the compounds was performed by measuring the melting point and gas chromatography coupled with mass spectrometry (GC–MS) on a Shimadzu GC–MS-QP system 2010SE. Synthetic compounds showed antimicrobial activity against Gram-positive cocci at MIC concentrations 0.16–80 μg/ml and Gram-negative bacilli at MIC concentrations 23.2–80 μg/ml. *Enterococcus faecium* and *S. aureus* had the best MIC values. The results of the cytotoxicity test indicated that the synthetic compounds showed no significant difference in three concentrations tested (5, 20, and 80 μg/ml), allowing cell viability not different from that assigned to the control, without the tested compounds. In this context, the development of DHPM derivatives brings an alternative and perspective on effectiveness of drugs as potential future antimicrobial agents.

## Introduction

Bacterial multidrug resistance is a serious and rapidly growing threat worldwide, leading to high morbidity and mortality rates (Tegos and Hamblin, 2014; Esposito and De Simone, 2017). Combating the advance of bacterial resistance to current antimicrobials should be a global priority.

It is estimated that in 2050, antimicrobial resistance will become one of the leading causes of death (Leung et al., 2011; O’Neill, 2016). It is thus crucial to discover new antimicrobials (Tacconelli et al., 2018).

This has increased the scientific interest in bioactive nitrogen-containing heterocyclic substances such as 3,4-dihydropyrimidin-2 (1H)-ones, or just dihydropyrimidinones (DHPMs). These compounds were first synthesized by the Italian chemist Pietro Biginelli in 1893 (Godoi et al., 2005; Mansouri et al., 2012; Venugopala et al., 2016). A pyrimidine ring integrates the molecular composition of various alkaloids (Prasad et al., 2016) and numerous nucleic acids (Kappe, 2000; Sharma et al., 2014).

There are reports of several pharmacological activities of analogues and derivatives of DHPMs, such as antitumor, antiviral, anti-inflammatory, antidepressant, antimalarial and anticancer (Ramachandran et al., 2016), antioxidant, antibacterial (Stefani et al., 2006; de Vasconcelos et al., 2012; Niemirowicz-Laskowska et al., 2018), insecticidal and larvicidal (Venugopala et al., 2016), and calcium channel modulation (Kappe, 2000; Fathima et al., 2013). Recent studies indicate effective action of pyrimidine analogs in the treatment of diabetes, by reducing the enzyme α-glucosidase and delaying the absorption of glucose (Bekircan et al., 2015; Peytam et al., 2021).

This present study evaluated *in vitro* the antimicrobial activity of three DHPM analogues against multiple drug resistant isolates from hospital patients.

## Materials and Methods

### General Procedure for the Synthesis of Compounds (4a–c)

**Figure 1:**
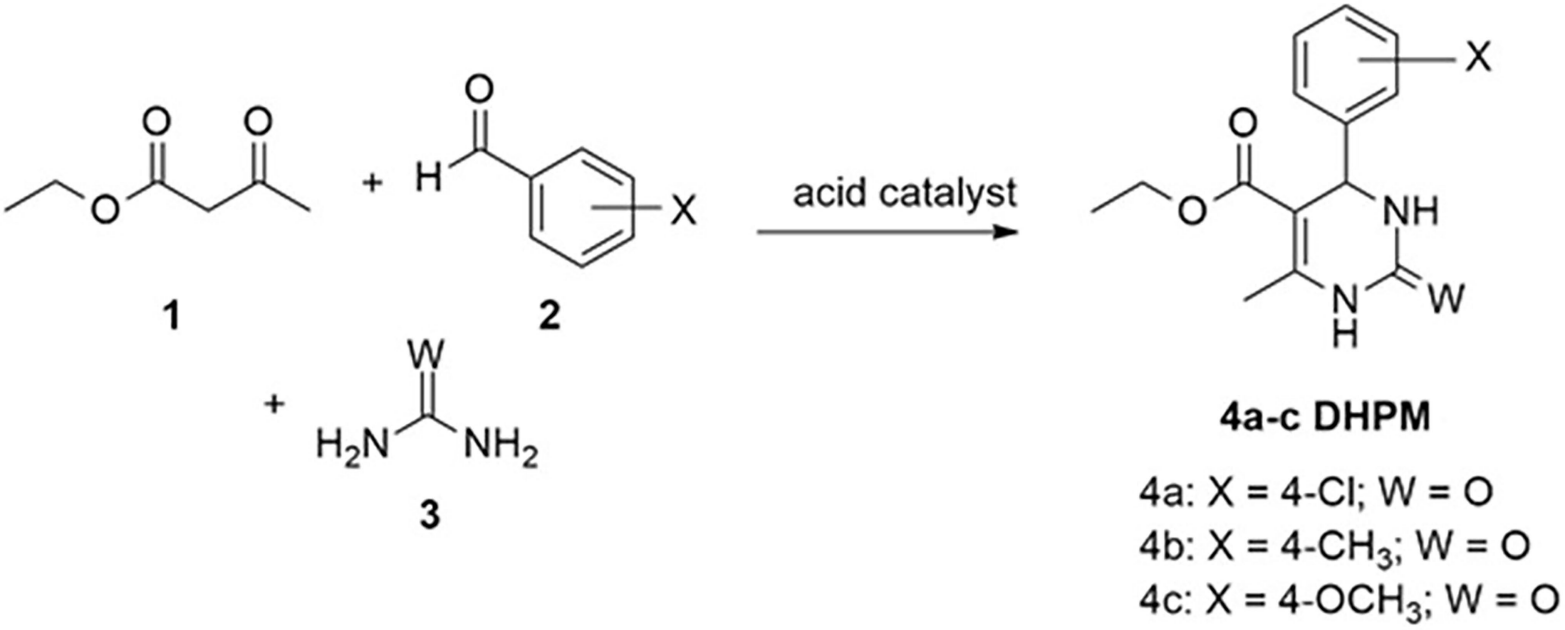
Biginelli reaction and formation of the compounds of interest.

The DHPMs were obtained in the Laboratory of Lipidomics and Bioorganics of Federal University of Pelotas, located in the state of Rio Grande do Sul, Brazil. The desired compounds were synthesized by mixing ethyl acetoacetate (1; 5 mmol), the appropriate aldehyde (2a–c; 5 mmoL), urea (3; 8 mmol), and citric acid (5 mmol) in 10 ml of absolute ethanol. The mixture was stirred under reflux for 4 h, according to de Vasconcelos et al. (2012), and the reaction’s progress was monitored by thin-layer chromatography (TLC) and gas chromatography (GC). The organic phase was extracted with ethyl acetate (2 × 10 ml), washed with cold water (2 × 20 ml), and dried with magnesium sulfate, and the solvent was removed under reduced pressure. The product obtained was purified by recrystallization with hexane and ethanol (Figure 1).

The chemical characterization of the compounds was performed by melting point measurement and gas chromatography coupled to mass spectrometry (GC–MS) in a Shimadzu GC–MS-QP 2010SE system.

### Multidrug-Resistant Bacterial Isolates

The bacterial isolates were provided by two hospitals in the city of Pelotas (here called Hospital A and Hospital B), collected from patients admitted between October 2018 and January 2019, consisting of samples of lung tissue, body fluids, skin, blood, and urine. The bacteria were previously identified as to species, and their resistance profiles were determined with the bioMerrieux VITEK 2 (Hospital A) and BD Phoenix (Hospital B) systems, according to the recommendations of the Clinical and Laboratory Standards Institute (CLSI), 2012. The effectiveness of DHPM was tested against three multi-resistant bacteria of each species, selected from the study by Jara et al. (2021).

Three previously identified isolates of each bacterial species were evaluated, namely seven Gram-negative bacilli (GNB): *Acinetobacter baumannii*, *Escherichia coli*, *Enterobacter cloacae*, *Klebsiella pneumonia*e, *Morganella morgannii*, *Pseudomonas aeruginosa* and *Serratia marcescens*; and two Gram-positive cocci (GPC): *E. faecium* and *S. aureus*, all of which showed resistance to at least three classes of antimicrobials. The bacterial resistance was tested previously by the hospitals against the following classes and antimicrobials: aminoglycosides (amikacin, gentamicin, and streptomycin); association with β-lactamase inhibitors (ampicillin/sulbactam, ampicillin/clavulanic acid, and piperacillin/tazobactam); carbapenems (ertapenem, imipenem, and meropenem); cephalosporins (cephalin, cefoxitin, cefuroxime, ceftriaxone, cefepime, cefotaxime, cefazolin, ceftazidime, and ceftaroline); glucopeptides (teicoplanin and vancomycin); glycylcyclines (tigecycline); lincosamides (clindamycin); macrolides (erythromycin); nitrofurans (nitrofurantoin); oxazolidinones (linezolid); penicillins (ampicillin, penicillin, and oxacillin); polypeptides (colistin); quinolones (nalidixic acid, ciprofloxacin, levofloxacin, moxifloxacin, and norfloxacin); rifampicin (rifampicin); and tetracyclines (minocycline).

This study was approved by the university’s research ethics committee under numbers 2,961,379 and 2,985,372 and by the National Ethics Committee on Research (CONEP) under number 2,880,831 (Brazilian Approval Platform). The experiments were carried out following biosafety standards and good laboratory practice (BRASIL, 2006).

### Minimum Inhibitory Concentration

The minimum inhibitory concentration (MIC) assay was carried out according to the guidelines of document M07-A9 from the Clinical and Laboratory Standards Institute (CLSI), 2012. A concentration of 1,600 μg/ml was obtained by weighing 4,800 μg of the DHPMs diluted in 3 ml of P.A. dimethylsulfoxide (DMSO). Then, a 1:10 dilution [0.5 ml of the DHPM compound solution with 4.5 ml of Müller-Hinton broth (MHB)] was obtained at the final concentration of 160 μg/ml. A 96-well sterile microplate was pre-filled with 100 μl of MHB in all wells, the first column was used as a negative control (with 100 μl of MHB), the second column was added 100 μl of the test compound dilution in the concentration of 160 μg/ml, obtaining a concentration of 80 μg/ml in this well, followed by 10 microdilutions in series until the penultimate well (column 11) reaching a concentration of 0.16 μg/ml, in the latter, 100 μl was removed and discarded. The last well (column 12) was used as a positive control (MHB added to the inoculum containing the microorganism of interest). To prepare the inoculum solution, the microorganism was seeded on blood agar plates for 24 h prior to testing in an oven at 37°C. A small part of the colony was removed and diluted in 3 ml of saline solution, until turbidity equivalent to the MacFarland scale 0.5 (1 × 10^8^ CFU/ml). Of this inoculum solution, 5 μl was added to each well from the second to the last well. Then, the plates were incubated at 37°C in an oven for 24 h. After this step, the MIC was evaluated by the colorimetric method with addition of 40 μl/well of the dye 2, 3, 5-triphenyltetrazolium chloride (TTC) at 0.015%. The plates were incubated in an oven at 37°C for 30 min. The visual reading involved the presence or absence of pink staining, which identified, respectively, metabolically active or inactive bacteria against the presence of the compound. The assays for each isolate were performed in duplicate and with three replicates on different dates.

### Minimum Bactericidal Concentration

After reading the MIC values, a test was performed to determine the minimum bactericidal concentration (MBC), through plating in brain heart infusion (BHI) agar. Aliquots of 5 μl from the well corresponding to the positive MIC (active bacteria) and the next well were collected. After plating in BHI, the plate was incubated at 37°C for 24 h to determine whether the concentrations were bactericidal or bacteriostatic. The method consists of observing the bacterial growth of the inoculum of the active bacteria of the respective MICs; where the growth of bacterial colonies defines in compounds with bacteriostatic action and non-bacterial growth defines the bactericidal action of the DHPM test compound.

### Cytotoxicity Assay

The cytotoxicity assay was performed in the Laboratory for Cell Biology and Tissue Center (NCT-BIO) of the School of Dentistry of Federal University of Pelotas. The cell viability assay was performed according to ISO 10993-5:2009 [International Organization for Standardization (ISO), 2009]. Mouse fibroblasts of the 3 T3 immortalized cell line (2 × 10-^4^/well) were cultured in Dulbecco’s Modified Eagle Medium (DMEM) supplemented with 10% fetal bovine serum (FBS), 2% L-glutamine, penicillin (100 U/ml) and streptomycin (100 mg/ml). Cells were incubated at 37°C in a humidified atmosphere of 5% CO_2_. The 3-(4,5-dimethylthiazol-2-yl)-2,5-diphenyltetrazolium bromide (MTT) assay (Sigma Chemical Company, St. Louis, MO, United States) was used to assess cell metabolic function by observing mitochondrial dehydrogenase activity.

The compounds were solubilized in DMSO and added to the DMEM medium, to obtain concentrations in the wells of 80, 20, and 5 μg/ml solubilized in 1.6% DMSO.

For evaluation of cell viability of the different DHPM analogues, the compounds diluted in 200 μl of DMEM were placed in the wells of 96-well plates containing mouse fibroblasts of the 3 T3 immortalized cell line (2 × 10-^4^/well). As a control, a group containing only fibroblast cells in DMEM was used. The plates were incubated for 24 h in a humidified atmosphere of 5% CO_2_. After incubation, the DMEM was removed and a MTT solution was placed in each well. After 4 h of incubation at 37°C in darkness, the blue formazan precipitate was extracted from the mitochondria using 200 μl/well of DMSO in a shaker for 5 min at 150 rpm. The absorption was determined using a spectrophotometer at a wavelength of 540 nm.

### Statistical Analysis

Statistical analysis of bacterial activity concentrations was obtained by the average of the samples tested. While one-way ANOVA was used to evaluate the difference between the treated groups. To confirm the significance of the differences between the concentrations of the compounds tested in relation to the control group (a group containing only fibroblast cells in DMEM), the Tukey *post hoc* test was used. The differences that presented *p* < 0.05 were considered as statistically significant.

## Results

### Chemical Data

The synthesized DMPM compounds 4a, 4b, and 4c have the following chemical characteristics.

4a. Ethyl 4-(4-chlorophenyl)-6-methyl-2-oxo-1, 2, 3, 4-tetrahydropyrimidine-5-carboxylate. Yield 90%; melting point 215°C; temperature: 215°C (Puri et al., 2009); GC–MS m/z, (%), observed: 295.05 [M + 1] (2.48%), 294.00 (14.65%), 265.00 (68.48%), 221.00 (43.70%), 183.10 (100.00%), 155.10 (53.68%), 137.05 (45.35%), 42.10 (43.11%). C14H15ClN2O3 [M] + required: 294.00.

4b. Ethyl 6-methyl-2-oxo-4-(p-tolyl)-1, 2, 3, 4-tetrahydropyrimidine-5-carboxylate. Yield 75%; melting point 216°C; temperature: 216–217°C (Debache et al., 2008) GC–MS m/z, (%), observed: 274.10 (16.08%), 245.10 (70.02%), 201.10 (53.35%), 183.10 (100.00%), 155.05 (51.91%), 137.05 (42.42%), 91.05 (26.01%), 42.05 (33.05%). C15H18N2O3 [M] + required: 274.13.

4c. Ethyl 4-(4-methoxyphenyl)-6-methyl-2-oxo-1, 2, 3, 4-tetrahydropyrimidine-5-carboxylate. Yield 91%; melting point 205°C; temperature: 204–205°C (Ranjith et al., 2010); GC–MS m/z, (%), observed: 290.10 (20.72%), 261.05 (100.00%), 217.10 (69.51%), 183.10 (55.24%), 155.05 (39.75%), 137.10 (36.14%), 42.05 (30.29%). C15H18N2O4 [M] + required: 290.13.

### Multidrug Resistant Bacterial Isolates

The bacterial isolates showed resistance to at least three classes of antibiotics, as shown by the susceptibility of the 15 classes tested (Table 1).

**Table 1:** Bacterial susceptibility profile of each class of antimicrobials tested in two hospitals in the city of Pelotas, Brazil, 2018–2019.

Bacteria/Isolates		Classes of antimicrobials^*^														
		Aminoglycosides	β-Lactamase inhibitors^**^	Carbapenems	Cephalosporins	Glucopeptides	Glycylcyclines	Lincosamides	Macrolides	Nitrofurans	Oxazolidinones	Penicillins	Polypeptides	Quinolones	Rifampicines	Tetracyclines
*Acinetobacter baumannii*	1	R	R	R	R	99	88	99	99	99	99	R	S	R	99	99
*Acinetobacter baumannii*	2	R	R	R	R	99	S	99	99	99	99	R	S	R	99	99
*Acinetobacter baumannii*	3	R	R	R	R	99	I	99	99	99	99	R	S	R	99	99
*Enterobacter cloacae*	1	R	R	R	R	99	R	99	99	99	99	R	S	R	99	99
*Enterobacter cloacae*	2	S	R	R	R	99	S	99	99	99	99	R	S	R	99	99
*Enterobacter cloacae*	3	R	R	S	R	99	88	99	99	99	99	R	88	R	99	99
*Escherichia coli*	1	S	S	S	R	99	88	99	99	S	99	R	88	R	99	99
*Escherichia coli*	2	R	R	S	R	99	S	99	99	99	99	R	S	88	99	99
*Escherichia coli*	3	R	S	S	R	99	88	99	99	99	99	R	88	R	99	99
*Klebsiella pneumoniae*	1	S	R	R	R	99	88	99	99	R	99	R	88	R	99	99
*Klebsiella pneumoniae*	2	S	R	S	R	99	88	99	99	R	99	R	88	R	99	99
*Klebsiella pneumoniae*	3	R	R	S	R	99	88	99	99	R	99	R	88	R	99	99
*Morganella morganii*	1	S	R	R	R	99	R	99	99	99	99	R	R	R	99	99
*Morganella morganii*	2	S	R	S	R	99	R	99	99	99	99	R	R	R	99	99
*Morganella morganii*	3	R	S	S	R	99	88	99	99	99	99	R	88	R	99	99
*Pseudomonas aeruginosa*	1	S	R	R	R	99	R	99	99	99	99	R	S	S	99	99
*Pseudomonas aeruginosa*	2	R	R	R	R	99	88	99	99	99	99	R	88	R	99	99
*Pseudomonas aeruginosa*	3	R	R	R	R	99	R	99	99	99	99	R	S	R	99	99
*Serratia marcescens*	1	S	R	R	R	99	R	99	99	99	99	R	R	R	99	99
*Serratia marcescens*	2	S	R	R	R	99	R	99	99	99	99	R	R	R	99	99
*Serratia marcescens*	3	S	R	S	R	99	88	99	99	R	99	R	88	S	99	99
*Enterococcus faecium*	1	S	88	99	88	R	R	R	R	99	S	R	99	R	88	88
*Enterococcus faecium*	2	88	88	99	88	R	88	88	88	S	S	R	99	R	88	S
*Enterococcus faecium*	3	88	88	99	88	R	88	88	88	S	S	R	99	88	88	R
*Staphylococcus aureus*	1	R	88	99	88	R	88	R	88	88	88	R	99	R	S	88
*Staphylococcus aureus*	2	88	88	99	88	S	88	S	R	88	S	R	99	88	S	R
*Staphylococcus aureus*	3	S	88	99	8	S	S	S	S	88	S	R	99	R	S	R

* *R: resistant; I: intermediate; S: sensitive; 88: not tested; 99: not applicable*.

** *β-Lactamase inhibitors: association with β-lactamase inhibitors*.

### Minimum Inhibitory Concentration

DHPMs demonstrated inhibitory potential against all bacterial species tested, inhibiting the growth of at least one isolate of each species. For BGN only bacteriostatic (inhibitory) activity was observed (concentrations from 23.3 to 80 μg/ml).

The MIC values were in the range of 0.16–80 μg/ml, with the lowest values referring to GPC, reaching 0.16 μg/ml, while attaining lower inhibitory activities for all multiresistant species of GNB, with MIC values from 23.2 to 80 μg/ml. Bacteriostatic activity was observed in *E. faecium* and *S. aureus* at DHPM concentrations ranging from 80 μg/ml to 0.16 μg/ml. The MIC values observed according to each bacterial species are described in Table 2 with the mean of the MICs.

**Table 2:** Mean of the minimum inhibitory concentration (MIC) of DHPM against multiresistant bacteria of hospital origin.

Bacterial species	^*^Compounds (μg/ml)								
	4a			4b			4c		
	Number of bacterial isolates			Number of bacterial isolates			Number of bacterial isolates		
	1	2	3	1	2	3	1	2	3
*A. baumannii*	40.00	40.00	80.00	64.00	60.00	50.00	70.00	66.70	60.00
*E. coli*	80.00	-	-	60.00	80.00	-	66.70	-	-
*E. cloacae*	80.00	80.00	53.30	80.00	80.00	60.00	66.70	80.00	-
*K. pneumoniae*	80.00	60.00	80.00	70.00	73.30	80.00	60.00	80.00	80.00
*M. morgannii*	80.00	80.00	60.00	60.0	80.00	66.70	56.00	70.00	23.30^**^
*P. aeruginosa*	60.00	70.00	56.00	80.00	60.00	80.00	80.00	53.30	60.0
*S. marcescens*	66.70	70.00	60.00	30.00^**^	-	-	30.00^**^	-	73.30
*E. faecium*	70.00	0.16^**^	0.16	80.00	33.40^*^	20.00	60.00	80.00	0.16
*S. aureus*	-	0.16	-	-	0.16	-	-	0.16	-

* *4a: 4-chlorophenyl; 4b: p-toluyl; 4c: 4-methoxyphenyl; (−) inconclusive MIC values*.

** *Values with statistically significant differences*.

The MIC values of the three compounds that showed simultaneous bacteriostatic activity for the same hospital bacterial isolate were submitted to ANOVA. In this analysis, only the isolate *S. aureus*^2^ was sensitive to the compounds, with no statistically significant difference between the values, similar to the finding described for *E. coli*^3^, which was not sensitive to any of the synthetic antimicrobials. *Morganella morgannii*^3^ showed a statistically significant difference in relation to compound 4c, which presented the best result for this species. For *S. marcescens*, compounds 4b and 4c showed a statistically significant difference in relation to compound 4a, so these two compounds were considered best for this species. In *E. faecium*^2^, DHPM 4a, followed by compound 4b, presented the best results.

Comparison of all the MIC values for the same species with the values according to the compounds, in order to obtain a value for each species, indicated that compound 4c had a statistically significant difference in relation to the others, thus being the compound with the best activity for *M. morgannii* when compared to *A. baumannii*, *K. pneumoniae*, *M. morgannii*, *P. aeruginosa*, and *E. faecium*. The results obtained in the present study were published in patent application number BR1020200216538 (Jara et al., 2021).

### Minimum Bactericidal Concentration

All DHPM analogues tested showed bactericidal activity in the GPC at concentrations that varied from 0.16 to 80 μg/ml. For GNB there was no bactericidal activity in any sample.

### Toxicity of the Synthetic Compounds

**Figure 2:**
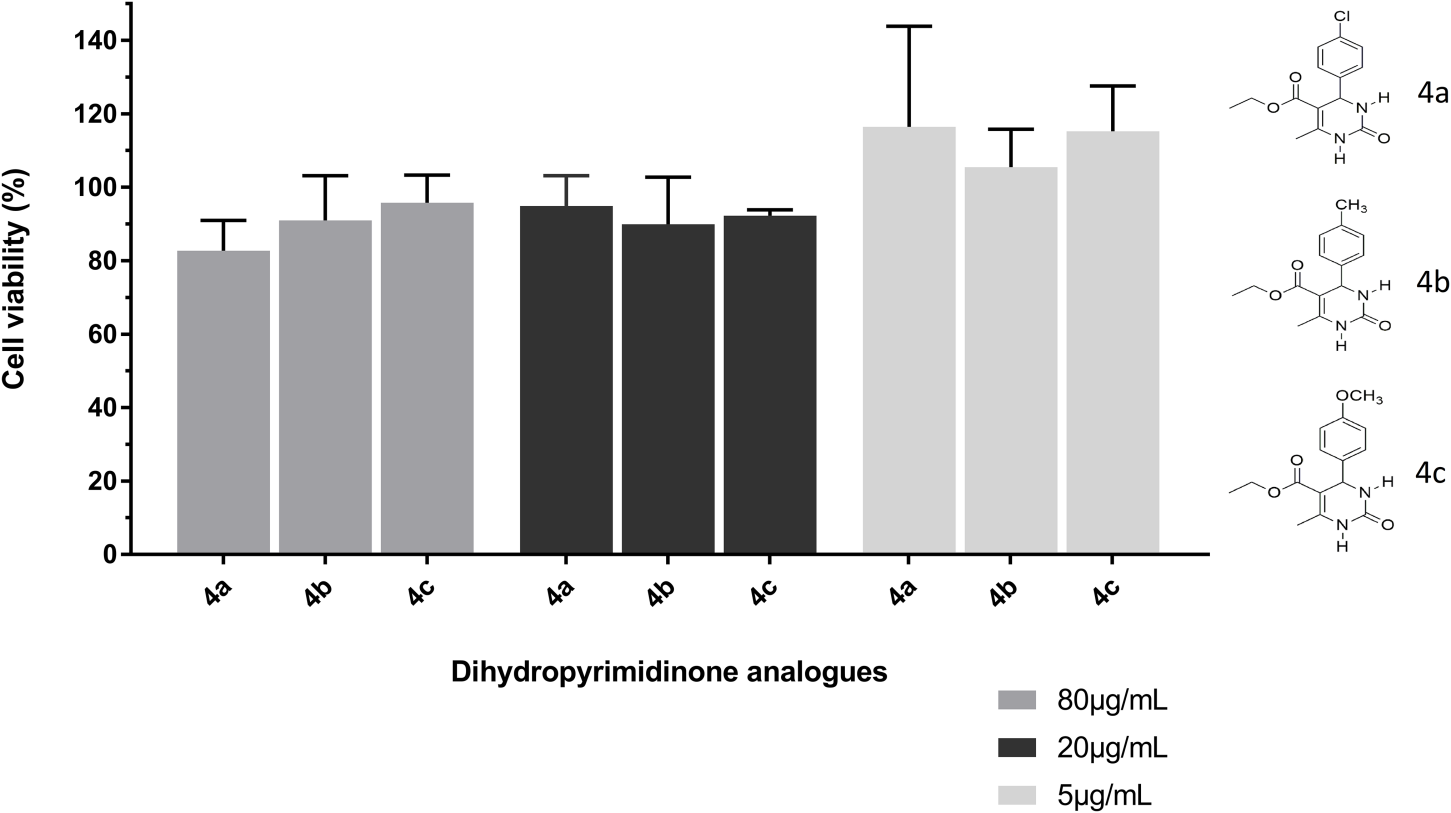
Cytotoxicity of dihydropyrimidinone analogs 4a (4-chlorophenyl), 4b (p-toluyl), and 4c (4-methoxyphenyl) using the MTT assay. The compounds were tested at concentrations of 80, 20, and 5 μg/ml and incubated for 24 h. The cell viability/proliferation using mouse fibroblasts of the 3T3 cell line (control = negative group representing cell viability of 100%) showed no difference among the groups (*p* > 0.05).

The three compounds were found to be non-cytotoxic to the cell lines at the concentration tested (80 μg/ml), as shown in Figure 1. The three compounds presented cell viability above 80% in the three concentrations tested (Figure 2).

The results of the cytotoxicity test indicated that the synthetic compounds showed no significant difference at the three concentrations tested (5, 20, and 80 μg/ml), allowing cell viability not different from that attributed to the control, without the presence of the tested compounds.

## Limitations

Our study had some limitations in testing the compounds with a small number of bacterial samples from each species, and it was relevant to analyze the cytotoxicity in other types of cells.

## Discussion

The problem of bacterial resistance to antimicrobials is multifaceted, from inappropriate drug management to a lack of investment in the discovery of new antimicrobials (Hughes and Karlén, 2014). Based on the definition by Magiorakos et al. (2012), the isolates used here are classified as multidrug-resistant, as they are resistant to three or more classes of antibiotics.

In the literature there are few reports of antibacterial activity in relation to the DHPM analogues described here, and we did not find any studies involving multidrug-resistant bacteria from hospitals. Chitra and Devanathan (2012) described analogues of DHPM with chlorine, nitrogen and fluorine at the 4-position of the aromatic aldehyde. These compounds showed *in vitro* antibacterial activity against *S. aureus*, *E. coli*, *K. pneumoniae*, *P. aeruginosa*, and *Salmonella typhi*.

Attri et al. (2017) and Medyouni et al. (2016) observed promising antibacterial activity of two of the same compounds analyzed by us (4a and 4b), but Attri et al. (2017) analyzed the compounds with the culture collection and gene bank of the Institute of Microbial Technology (Chandigarh, India) and Medyouni et al. (2016) analyzed standard bacterial strains. While in our study, we tested these compounds against multidrug-resistant hospital bacteria.

Attri et al. (2017) tested the DHPM compounds against the bacterial strains *E. coli* (MTCC 443), *S. aureus* (MTCC 3160), *P. aeruginosa* (MTCC 2581), and *K. pneumoniae* (MTCC 7028). Their results indicated that compound 4a showed inhibitory activity against all strains tested, while 4b did not show antibacterial activity against the *E. coli* and *K. pneumoniae* strains.

In our study, we used concentrations in μg/mL and Attri et al. (2017) used units in ppm, with equivalent concentrations (1 ppm = 1 μg/ml). According to Attri et al. (2017), compound 4a showed results against *E. coli* between 31.250 and 15.625 ppm, showing higher inhibitory activity in relation to our findings, which ranged from 60 to 80 ppm. (μg/ml). Regarding *S. aureus*, the authors reported moderate activity, with MICs of 62.5–125.0 ppm of compounds 4a and 4b, while the MIC was 0.155 ppm (μg/ml), indicating excellent antibacterial activity. Against *P. aeruginosa*, compounds 4a and 4b showed good antibacterial activity, with MIC values in the range of 15.625–31.250 ppm (μg/ml), while our tests of compound 4b showed 60 to 80 ppm (μg/ml), and of compound 4a resulted in 56 to 70 ppm (μg/ml). Therefore, Attri et al. (2017) obtained more promising results than our study. For compound 4a, Attri et al. (2017) reported activity against *K. pneumoniae* bacteria only with MICs of 31.25–62.50 ppm (μg/ml), while we found MICs between 60 and 80 ppm (μg/ml), Both studies showed very similar results at this point.

Medyouni et al. (2016) tested the efficacy of compound 4b *in vitro* by the plate diffusion method against standard GPC strains (*Agrobacterium tumefaciens*, *Listeria monocytogenes* ATCC 19117, *Micrococcus luteus* LB 14110 and *S. aureus* ATCC 6538), and GNB (*Salmonella Typhimurium* ATCC 14028 and *P. aeruginosa* ATCC 49189). The authors observed MIC values of 2.5 mg/ml (2,500 μg/ml) and 0.016 mg/ml (16 μg/ml) for *S. aureus* and *P. aeruginosa*. However, it is not possible to compare their results with our experimental application due to the different techniques used by those authors.

Ramachandran et al. (2016) evaluated *in silico* the antimicrobial activity of DHPM analogues and suggested that this class of compounds may be important to overcome the problem of bacterial resistance to antimicrobials.

Bacteriostatic agents include tigecycline, linezolid, macrolides, sulfonamides, tetracyclines and streptogramins, while bactericidal agents include β-lactams, glycopeptides, fluoroquinolones and aminoglycosides (Nemeth et al., 2015). Although it seems preferable for an antibiotic to kill bacteria rather than just inhibiting them, there are few reports that the clinical importance of a bactericidal action observed *in vitro* is better than a bacteriostatic action (Rhee and Gardiner, 2004). Studies suggest that combinations of bactericidal and bacteriostatic agents may lead to better clinical outcomes compared to single use. However, there are diseases such as endocarditis and meningitis where clinical experience favors the use of bactericidal agents (Finberg et al., 2004). Clindamycin and chloramphenicol are bacteriostatic antibiotics that slow bacterial growth, usually by inhibiting protein synthesis or reducing cellular respiration. As a result, the infectious agent is more easily eliminated by the immune system (Bernatová et al., 2013; Lobritz et al., 2015).

In the context of the emerging need to discover new products with antifungal and antibacterial properties, the development of DHPM derivatives is an alternative for more effective future antimicrobial agents. The results of *in vitro* antibacterial activity suggest that compounds A, B and C have potent *in vitro* antibacterial activity against multidrug-resistant hospitals bacteria. Furthermore, the cytotoxicity study revealed that all compounds did not show significant cytotoxicity against mouse fibroblast cell lines at the highest concentration evaluated, indicating the selectivity of their antimicrobial action. All three compounds showed antibacterial activity against both GNB and GPC.

Thus, it is necessary to continue research into the antimicrobial potential of these compounds, as well as to elucidate the mechanism of action attributed to them.

## Conclusion

In the context of the emerging need to discover new products with antifungal and antibacterial properties, the development of DHPM derivatives brings an interesting alternative and perspective on the efficacy of drugs as future antimicrobial agents. The results of *in vitro* antibacterial activity suggest that compounds A, B, and C have potent *in vitro* antibacterial activity in multiresistant bacteria of hospital origin. In addition, the cytotoxicity study revealed that all compounds did not show significant cytotoxicity against mouse fibroblast cell lines at the maximal concentration assessed, indicating the selectivity of their antimicrobial action. All three compounds showed antibacterial activity in both BGN and CGP. Thus, it is necessary to invest in the continuity of research concerning these compounds.

## Data Availability Statement

The raw data supporting the conclusions of this article will be made available by the authors, without undue reservation.

## Author Contributions

MC conceived and designed the study and was responsible for the data curation and analysis of the experiments. MC and AA performed the statistical analysis. MR and LS wrote and reviewed different sections of the manuscript. AS was responsible for the formal analysis of the cytotoxicology section and performed the statistical analysis. CL and PS contributed to the laboratory analysis of the experiments. CM was responsible for the resources material and the synthesis and supply of compounds. PN managed the project, its resources, and validation. All authors contributed to and commented on the manuscript text and approved its final version.

## Funding

This study was partially financed by the Coordination for the Improvement of Higher Education Personnel (CAPES – Brazil), financial code 001. PS received a grant from CAPES (no. 88887.502085/2020-00). LS received a grant from the Fundação de Amparo à Pesquisa do Estado do Rio Grande do Sul (FAPERGS), no. 124733/2019-0. This study was also funded by the National Program for Quality Control (PNCq), no. 03/2018, and the Institutional Scientific Initiation Scholarship Program (PIBIC).

## Conflict of Interest

The authors declare that the research was conducted in the absence of any commercial or financial relationships that could be construed as a potential conflict of interest.

## Publisher’s Note

All claims expressed in this article are solely those of the authors and do not necessarily represent those of their affiliated organizations, or those of the publisher, the editors and the reviewers. Any product that may be evaluated in this article, or claim that may be made by its manufacturer, is not guaranteed or endorsed by the publisher.
